# Does cultural distance energize employees? The moderating role of psychological safety

**DOI:** 10.1371/journal.pone.0252406

**Published:** 2021-06-01

**Authors:** Yingjie Yuan

**Affiliations:** Faculty of Economics and Business, University of Groningen, Groningen, The Netherlands; University of Education, PAKISTAN

## Abstract

The increasingly globalized workforce and the growing need for boosting employee energy have engendered both practical and research interest in stimulating employee energy in intercultural interactions. Yet neither the culture research nor the energy literature has explored the link between cultural distance and employee relational energy—the heightened level of psychological resources in social relations. This paper presents empirical evidence of cultural distance stimulating relational energy. Further, building upon the threat-rigidity theory, I propose that cultural distance stimulates relational energy more when employees perceive high levels of psychological safety. Two studies were conducted to test these two hypotheses. One laboratory experiment on 202 international students at a Dutch university provided causal evidence of the positive relationship between cultural distance and relational energy. Next, a two-wave field study on 373 international employees was conducted to replicate this main effect of cultural distance and further investigate the moderating role of psychological safety. Results supported that employees with higher levels of psychological safety are more prone to experience enhanced relational energy as a result of cultural distance. These findings contribute to the scarce research on possible positive influence of cross-cultural communication at work, and also advance the growing research on the antecedents of employee relational energy. The implications for practitioners to energize employees are also discussed.

## Introduction

Organizations are more than ever confronting the challenge of managing the intercultural workforce. The prevalent intercultural collaboration at work draws research attention to the consequences of cultural distance–the similarities and dissimilarities in cultural views, norms, and beliefs [1]. Myriads of studies have evidenced cultural differences in employee behaviours and communication [2]. It is thus important to investigate how employees respond to the impact of cultural distance at work. Nevertheless, as stressed in a recent review, the disproportionate focus (15: 1) on the dark side of cultural distance hampers the balanced development of this literature [3]. Very little attention was paid to how cultural distance affects employee wellbeing such as positive affect, engagement, and energy. Understanding the link between cultural distance and employee wellbeing from the perspective of Positive Organizational Scholarship (POS) is pending.

From the lens of POS, relational energy is one of the most fundamental resources for employee performance [4], creativity [5], job satisfaction [6], and engagement [7]. Relational energy refers to a heightened level of psychological resourcefulness from social interactions that help employees do their work [7]. This construct reflects one’s capacity for actions as a result of the activation of vigour, enthusiasm, vitality, etc. Given the positive role of relational energy for organizational outcomes and the fact that employees nowadays tend to experience declined energy and more stress at work, it is important to understand what cultivates employee relational energy. To understand the sources of relational energy, researchers have primarily focused on individual differences such as extraversion [8] and humility [9]. The influence of relational contexts, however, remains under the radar [10, 11]. Examining employee wellbeing in intercultural contexts has been underscored for years [12]. Yet there are few empirical endeavours addressing this question to date [10]. It is thus important to investigate the influence of cultural distance on employee relational energy.

This study aims to explore the link between cultural distance and employee relational energy. Relational energy in essence is a type of psychological resource. People seek psychological resources from their social environments, and consume them to meet job demands. Some interactions inspire and excite employees [13], whereas others deplete psychological resources and are de-energizing [14]. Cultural distance highlights interpersonal differences in verbal and non-verbal communications [15], social responses [16], supportive behaviours [17], and conflict resolution [18]. Such differences in intercultural interactions presumably foster or consume psychological resources from different perspectives.

Existing literature from different perspectives suggests two different readings of this relationship. The intergroup literature seems to imply a negative link between cultural distance and relational energy, because high cultural distance may trigger social categorization, which reduces intergroup contacts and generates intergroup distress and threats [19]. Moreover, people tend to be less sensitive to emotional signals in intercultural communication [20]. Cultural distance may thus deplete psychological resources and hinder the transmission of relational energy.

Nevertheless, there is also evidence implying a positive impact. Due to the unique values and norms from other cultures, people may find different cultures attractive and seek intercultural contacts [21]. Prior studies also showed that cultural distance relates to higher satisfaction and creativity in teams [22], and lower intercultural conflicts [23]. In this line, such excitement and attractive stimuli in intercultural contacts may provide psychological resources for relational energy. Therefore, I propose two competing hypotheses regarding the relationship between cultural distance and employee relational energy.

***Hypothesis 1a*.** *Cultural distance has a negative influence on employee relational energy*.
***Hypothesis 1b*.** *Cultural distance has a positive influence on employee relational energy*.

The relationship between cultural distance and employee relational energy reflects how employees respond to the new stimuli brought by different cultural backgrounds. Focusing on how organizations and individuals respond and react to environmental threats, the threat-rigidity theory provides an integrative frame to elucidate these competing readings [24, 25]. It suggests that, when feeling safe, people could adapt and appreciate environmental challenges such as different cultures. But when perceiving threats, individuals tend to respond rigidly. This perspective provides a perfectly fitting lens to explain the influence of cultural distance on employee relational energy. Further, it points to the moderating role of psychological safety–the perceptions of to what extent the interpersonal context is safe for risk tasking [26]. Psychological safety encourages people to open up to others, stimulate more social contacts [27], and actively speak up [28]. When feeling safe in intercultural contexts, employees are more susceptive of positive psychological resources and are more likely to experience heightened relational energy. In contrast, low psychological safety implies risk-aversion, and leads to more conflicts and distress in interpersonal contexts [29]. It obstructs the development of relational energy in intercultural interactions. Low psychological safety also intensifies the depletion of psychological resources. Therefore, I propose:

***Hypothesis 2*.** *Psychological safety moderates the relationship between cultural distance and employee relational energy*, *such that cultural distance is more likely to heighten employee relational energy when employees experience high levels of psychological safety*.

**Fig 1:**
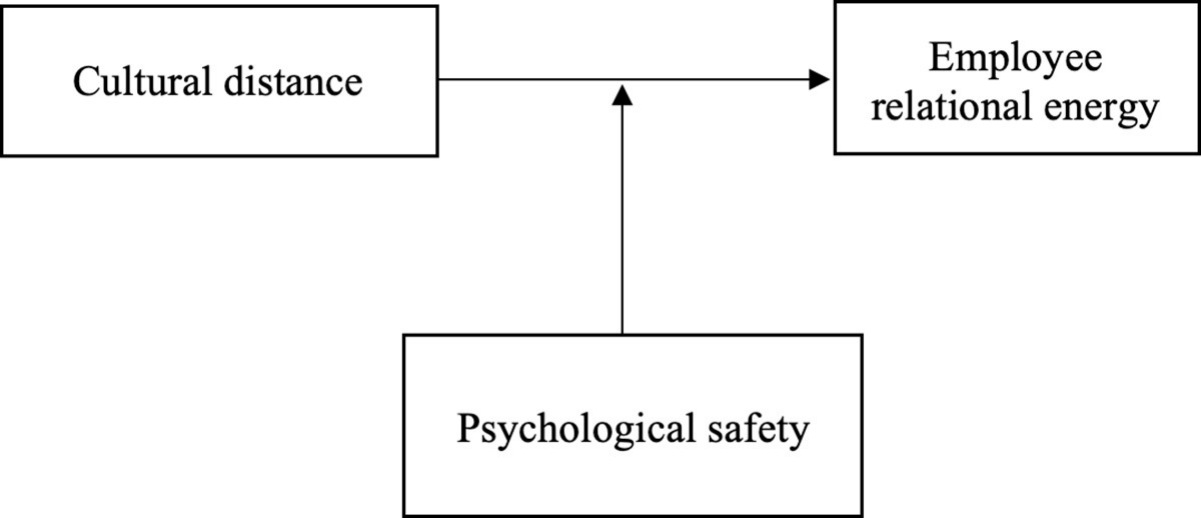
Conceptual model of cultural distance and employee relational energy.

I tested the hypotheses in two studies (see Fig 1 for the conceptual model). Study 1 employed an experimental design to provide causal evidence for the main effect. Specifically, I manipulated cultural distance and observed the development of relational energy in a dyadic task. Study 2 examined both hypotheses with a two-wave sample of international employees across cultures.

## Study 1: Methods

### Participants and procedure

A convenience sample of 202 students with diverse cultural backgrounds in a Dutch University participated for course credits or monetary rewards (8 euro per hour). 48.5% were female students. Average age was 21.45 (*SD*_*age*_ = 2.60).

Two research assistants scrutinized the cultural origins of participants and randomly matched them in pairs with different cultural distances (control vs. low vs. high). Upon arrival, participants followed instructions to work together on the survival task of Lost at Sea, which has been widely used to create collective work experiences. After this task, participants answered an online survey individually, and were then debriefed and thanked.

This study was approved by the University of Groningen Research Ethics Committee from the Faculty of Economics and Business (Ref: FEB-20180820-7260). All participants provided written informed consent. The raw data is available at https://osf.io/3yrjt/.

### Manipulation

*Cultural distance* was manipulated based on the cultural clustering [30]. They clustered national cultures into cultural groups based on their similarities scoring on Hofstede’s four cultural dimensions. Cultures from adjacent clusters (e.g., the Netherlands and Denmark) are considered less distant than those in distant clusters (e.g., the Netherlands and India). I manipulated three levels of cultural distance (control vs. low vs. high). The control condition consisted of 32 pairs of Dutch-Dutch students; the low-distance condition had 34 Dutch-European pairs (e.g., Dutch and Danish); and the high-distance condition included 35 Dutch-Asian pairs (e.g., Dutch and Indian).

#### Manipulation check

For rigorism, two research assistants verified the cultural origins of participants upon their arrival. Students reporting inconsistently (e.g., confusing ethnicity with nationalities) were turned down. This guaranteed a successful manipulation.

### Measures

*Relational energy* was measured with the five-item inventory from [7] on a 7-point scale (1 = “strongly disagree”, 7 = “strongly agree”). One sample item was “*After interaction with my task partner*, *I feel more energy to do my work”*. Cronbach’s alpha was .80.

### Study 1: Results

Table 1 reports the descriptive statistics.

**Table 1:** Descriptive statistics in Study 1.

	Relational energy (Y)		
	$\bar{Y}$	*SD*	${\bar{Y}}^{*}$
Control condition (*n* = 64)	4.344	.807	4.368
Low-distance condition (*n* = 68)	4.579	.897	4.551
High-distance condition (*n* = 70)	4.671	.938	4.676
All groups combined	4.537	.890	4.532

*N* = 202.

As shown in Table 2, results supported the positive effect of cultural distance on relational energy (*B* = .16, *SE* = .08, *p* = .04, *95%CI* = [.01, .32]). Compared against the control group, the low-distance condition did not significantly differ (*B* = .24, *SE* = .15, *p* = .12); but the high-distance condition significantly improved (*B* = .33, *SE* = .16, *p* = .03). This supported Hypothesis 1b.

**Table 2:** Regression results of Hypothesis 1 & 2 in Study 1 & 2.

	Model 1			Model 2		
	*B*	*SE*	*95% CI*	*B*	*SE*	*95% CI*
*Dependent variable*: *employee relational energy (Hypothesis 1*, *Study 1)*						
Constant	4.21***	0.17	[3.87, 4.54]			
Cultural distance	0.16*	0.08	[0.01, 0.32]			
*R*^*2*^*(ΔR*^*2*^*)*	.02*					
*Dependent variable*: *employee relational energy (Hypothesis 1*, *Study 2)*						
Constant	4.52***	0.07	[4.38, 4.66]			
Cultural distance	0.14*	0.07	[0.00, 0.28]			
*R*^*2*^*(ΔR*^*2*^*)*	.01*					
*Dependent variable*: *employee relational energy (Hypothesis 2*, *Study 2)*						
Constant	4.52***	0.07	[4.39, 4.65]	4.51***	0.06	[4.38, 4.63]
Cultural distance	0.10	0.06	[-0.03, 0.23]	0.12^†^	0.07	[-0.01, 0.25]
Psychological safety	0.44***	0.07	[0.31, 0.56]	0.468***	0.07	[0.34, 0.60]
Cultural distance × Psychological safety				0.17**	0.07	[0.04, 0.30]
*R*^*2*^*(ΔR*^*2*^*)*	.15***			.17***(.02)		

*N* = 202 (Study 1). *N* = 283 (Study 2).

*** *p* < .001

** *p* < .01

* *p* < .05

^†^ *p* < .05.

## Study 2: Methods

### Sample and study design

Study 2 tested both hypotheses with a convenience sample of international employees with at least one year of full-time work experience through the Prolific Academy platform. I recruited employees with at least two months of intercultural collaboration experience at work. A two-wave design was used to capture the development of relational energy. 344 qualified employees participated in the wave-1 survey. Three weeks later, 309 participants completed the wave-2 survey. After cleaning invalid responses (e.g., failed attention checks), I retained 283 valid responses. The response rate was 91.6%.

Participants were from 23 cultures. They reported in this survey their interactions with co-workers from 54 distinct cultures. 51.9% were female. 74.2% had a bachelor’s degree and above. The average age was 39.33 (*SD*_*age*_ = 11.02). This sample also featured diverse job positions, with 48.8% of first-line employees, 39.9% of middle management, 7.4% of senior management, and 3.9% of top management.

This study was approved by the University of Groningen Research Ethics Committee from the Faculty of Economics and Business (Ref: FEB-20200224-10470). All participants provided written informed consent. The raw data is available at https://osf.io/6a2q5/.

### Measures

#### Cultural distance

Participants reported cultural origins for themselves and their co-workers at wave 1. Cultural distance was computed as the Euclidean distance index on Hofstede’s four cultural dimensions. Cross-culture studies have suggested the superiority of this measure because it does not assume equal weights of different cultural dimensions [31]. The algorithm follows:

$${CD}_{ij}=\sqrt{\sum_{k=1}^{4}{\left(I_{ki}-I_{kj}\right)}^{2}}$$

where *CD*_*ij*_ denotes the Euclidean distance index between country *i* and country *j*; *i*_*k*_ and *j*_*k*_ are the Hofstede’s cultural scores on the cultural dimension *k* (from 1 to 4) for country *i* and country *j* respectively.

*Psychological safety* was self-reported at wave 1, with the seven-item inventory from [26] on a 7-point Likert scale (1 = “*strongly disagree*”, 7 = “*strongly agree*”). A sample item was “*It is safe for me to take a risk when interacting with this colleague*”. Cronbach’s alpha was .84.

*Relational energy* was self-reported at wave 2 with the same scale used in Study 1. Employees were instructed to refer to their intercultural interactions when answering questions. Cronbach’s alpha was .93.

### Study 2: Results

Table 3 reports descriptive statistics of variables in Study 2.

**Table 3:** Means, standard deviations, and correlations in Study 2.

Variables	*M*	*SD*	1	2	3
1. Relational energy	4.52	1.18	(.93)		
2. Cultural distance	2.04	1.14	.12*	-	
3 Psychological safety	5.41	0.95	.38**	.09	(.84)

*N* = 283.

**p < .01

* *p* < .05.

**Fig 2:**
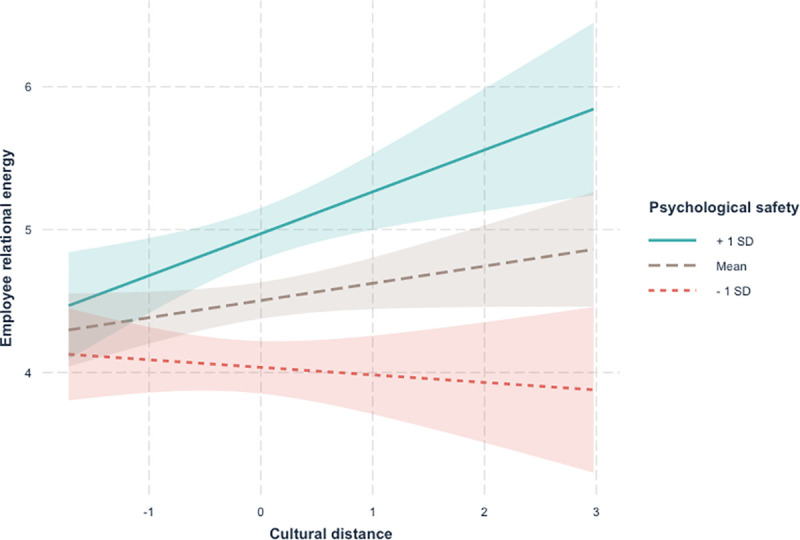
Interaction between cultural distance and psychological safety in Study 2.

Consistent with Study 1, results supported the positive impact of cultural distance on employee relational energy (*B* = .14, *SE* = .07, *p* = .05). Hypothesis 1b was supported. In support for Hypothesis 2 (see Table 2), results showed a positive interaction between cultural distance and psychological safety (*B* = .17, *SE* = .067, *p* = .01), with the interaction term explaining 2% of the variance (F [1, 278] = 6.74, *p* = .01). The entire model explained 16.81% of the variance. Further, analysis of the regions of significance suggested that, cultural distance positively related to employee relational energy at moderate-to-high levels (0.05 SD and above) of psychological safety (see Fig 2), and negatively at low levels (-3.05 SD and below) of psychological safety.

## Discussion

Cultural distance at work was often considered as a communication barrier and a dark influence on interpersonal relations [3]. Since employees experience job burnouts and lowered energy at work more than ever in the globalized world, organizations on one hand strive to optimize intercultural communication and on the other hand endeavor to cultivate employee energy at work. It is therefore vital to understand the influence of cultural distance on employee relational energy. To that end, I applied the threat-rigidity theory to explain the influence of cultural distance on employee relational energy and further propose psychological safety as a boundary condition. In support of this moderation model, I found that cultural distance tends to boost the relational energy that employees experience in intercultural interactions (Hypothesis 1b), and that psychological safety positively moderates this relationship between cultural distance and employee relational energy (Hypothesis 2). Employees experiencing higher levels of psychological safety at work are more prone to feel energized by cultural distance in interpersonal communication.

### Theoretical implications

Fostering relational energy in cross-cultural interactions at work is of great importance in the globalized business environment [32]. Moving from decades of research on the dark side of cultural distance, recent studies have increasingly called for empirical attention to the positive implications of cultural distance for employee wellbeing and health [3]. This article presents the first empirical evidence of cultural distance fostering employee relational energy, particularly for people with high psychological safety. This inspires further investigations on the positive impact of cultural differences in social contexts. One future research agenda can focus on linking cultural distance with other positive outcomes such as job engagement and innovation [33]. How cultural distance promotes relational energy is also a valuable question. Besides, the findings of this article advance the growing literature on employee energy. Energy scholars have not yet examined relational contexts as the antecedents of employee energy [5]. Supportive evidence for the positive impact of cultural distance invites future studies to explore social dynamics such as status differences.

### Practical implications

The value of boosting employee relational energy is salient. Positive experiences of relational energy are associated with employee wellbeing, job engagement and satisfaction, and job performance [7, 9]. In the time of the Covid-19 pandemic where employees generally suffer from declined mental health [34], it is of particular importance for organizations to concern themselves about employee energy at work and to take measures to improve their psychological states. Given that many organizations nowadays are involved in cross-cultural communication and global assignments to different extents, the present article reveals a simple yet effective intervention of creating intercultural contacts at work to energize employees. The findings of two studies support that, interacting with colleagues from distant cultures is likely to bring positive energy for employees to engage in their tasks and feel vigorous at work. Monitoring and addressing the positive impact of cultural distance on relational energy would therefore add value to not just multinational firms of employees with diverse cultural backgrounds, but also organizations that deal with intercultural business operations and expatriates on global assignments. As this positive influence exists for people with moderate-to-high psychological safety, policymakers and managers are advised to monitor and stimulate psychological safety of the intercultural workforce, such as expatriates and global project teams.

### Limitations

This study is not without limitations. First, although Study 2 measured relational energy and psychological safety in different waves, future studies are recommended to collect multi-source data to eliminate common-source biases. Second, both studies sampled mainly from western cultures. It is valuable to test this model on other continents for generalizability.
